# Urethral hemangioma: case report and review of the literature

**DOI:** 10.11604/pamj.2016.23.96.8700

**Published:** 2016-03-15

**Authors:** Souhail Regragui, Amine Slaoui, Tarik Karmouni, Khalid El Khader, Abdelatif Koutani, Ahmed Ibn Attya

**Affiliations:** 1 Urology B, Ibn Sina University Hospital, Rabat, Morocco

**Keywords:** Urethral hemangioma, woman, surgery

## Abstract

Hemangiomas are benign vascular tumors. They are the prerogative of the liver and skin. And genitourinary localizations are rare and have only been rarely reported in the prostat, bladder, ureter or the perineum. In the light of published cases, urethral hemangiomas are mostly found in males. Few cases of hemangioma in the female urethra were reported. We report a cavernous hemangioma of the urethra in a 61 years old patient who presented bleeding from the urethra and micturition disorders. Physical examination revealed a tumor 3 cm x 2 polyploid arising from the terminal urethra (urethral hemangioma). We performed surgical resection of the tumor, along with bladder drainage. The postoperative course was simple. We update through a review of the literature aspects of the diagnostic and therapeutic care of the urethral hemangioma.

## Introduction

Hemangiomas are benign vascular tumors. They are the prerogative of the liver and skin. Genitourinary localizations are rare and have only been rarely reported in the prostat, bladder, ureter or the perineum [1]. We report a cavernous hemangioma of urethra in a 61 years old woman in view of its rarity. From this observation, we present the clinical and pathological aspects and report our therapeutic strategy.

## Patient and observation

**Figure 1:**
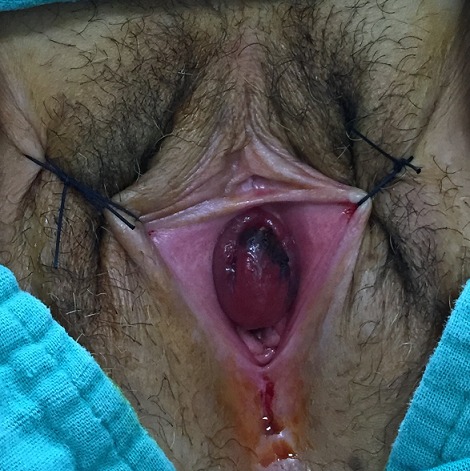
Polypoid mass arising from the terminal urethra

**Figure 2:**
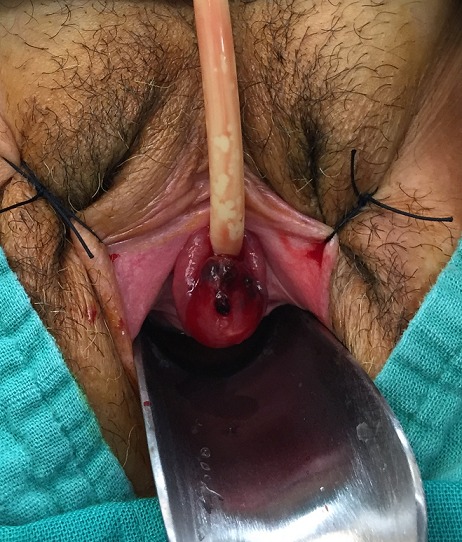
Preoperative appearance with the urinary catheter

A 61 years old woman with no particular medical history consulted for urethral mass causing dysuria and intermittent urethral bleeding since 2 months. Physical examination revealed a vascularized tumor which depended on the lower commissure of the urethral meatus of approximately 3 x 2 cm (Figure 1). The biological assessment was correct: no anémia or electrolyte disorder or blood dyscrasias disorder. Ultrasound sonography abdomen-pelvis showed no significant pathology. The urethrocystoscopy showed that the tumor was prolonged until the first third of the urethra and easily bleeding. Finally, cystoscopy was able to cross the tumor and to explore the posterior urethra, bladder neck, and bladder wich appeared normal. The patient has undergone surgical resection of the tumor and a bladder drainage was set up for a period of 3 days (Figure 2). Histopathological study reported a urethral hemangioma with large venous vessels dilated between a lax stroma without atypia cytonucléaire. The vessels are filled with red blood cells. They are sometimes pre thrombosis evidenced by the existence of fibrin deposits. Between vessels, are some beams regular smooth muscle cells. The postoperative course was simple and to this day there has been no recurrence.

## Discussion

**Figure 3:**
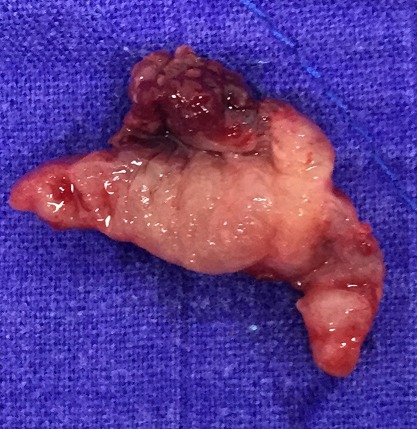
The excised tissue

Hemangiomas are benign vascular tumors that can occur at any age but it seems to be a male predominance [2]. They are the prerogative of the skin and liver. However, they can grow at all levels of the urinary tract, including kidney, ureter, bladder, prostate and urethra. The latter location may be less frequent. Tumors can be single or multiple [3]. Clinical symptoms depend on the location and size of the tumor. The main symptom is the presence of microscopic bleeding whether or macro haematuria, urethral bleeding or hematospermia. Besides this bleeding can be very intense and cause anemia [1]. In our case, the urethrorrhagya didn't cause any anemia, nevertheless it was the main symptom that leads the patient to consult. The pathogenesis is not entirely understood, two hypotheses clash. Some defend the embryological origin that it follows from the rest of embryonic angioblastiques unipotent cells fail to develop into normal blood vessels [4]. However, it can not be excluded that it could also be due to degenerative processes associated with chronic irritative factors and atrophic. The latter hypothesis would explain that the tumor can occur at a relatively advanced age, as is the case of our patient. Thus, this clinical case seems to refute the embryological thesis. Therefore, It may co¬exist with external hemangiomas and con¬genital disorders like Sturge Weber or Klippel-Trenaunay Weber syndrome [5]. The urethrocystoscopy has both diagnostic and therapeutic purposes. Indeed, it allows the identification of tumor characteristics, friability, its size, location and number. Moreover it facilitates the selection of therapeutic strategy [6]. In our case it was a single tumor which depends of the lower commissure of the urethral meatus. Its size was approximately 3 x 2 cm. Among the differential diagnosis of urethral hemangioma include wattles of the urethra, polyps, fibroids, periurethral abscess, but it is important not to disregard the malignant tumors, such as adenocarcinomas, cell tumors transitional, sarcomas, melanomas and squamous cell carcinomas [1]. The treatment consists of a tumor resection or ablation. Often hemangiomas are underestimated can appear deceptively smaller. This is because of a possible deep implantation in the inner layers of the urethra. Complete resection could be so laborious and recurrences are common [7]. Treatment of urethral hemangiomas can be based medical oral steroids, such as endoscopic electrocautery or laser ablation, cryotherapy, or open surgery as in our case [8] (Figure 3). Furthermore, selective arterial embolization has been reported [9]. One could even use a urinary diversion in some cases [10]. In our case, the patient kept the vesical probe three days. The postoperative course was simple. The patient hasn't presented any recurrence.

## Conclusion

Simple transurethral excision of small urethral hemangiomas may be an effective treatment especially when laser facilities are not available. The cornerstone of the right management of urethral hemangiomas is still careful preoperative definition of the extension of the lesion.
